# Progress in Dermatology

**Published:** 1895-02-23

**Authors:** 


### Progress in Dermatology.

**Eczema.**—A discussion on the management of eczema at the British Medical Association was opened by Morris,[1] who discussed the following points in relation to eczema: With regard to internal treatment, he considered that the less internal medication there is, in eczema, the better for the patient. If a constitutional dyscrasia underlies the skin affection, it must of course be treated on the ordinary principles of medicine. With regard to the influence of diet, the author stated that it has no influence at all except indirectly. With regard to local treatment, every case should be treated as if it were of parasitic origin; the first object is to destroy the micro-organisms; the second, to protect the inflamed surface from the air and from fresh microbic invasion; and the third, to soothe the irritation.

The tendency to the recurrence of eczema in predisposed persons is overcome by change of climate, especially if accompanied by complete rest of mind as well. He did not consider that any natural waters had any specific action on eczema, and that the sulphur waters had no other effect than that due to their anti-parasitic action.

Unna,[2] at the same meeting, demonstrated a series of microscopic sections of chronic eczema, in which micrococci were well seen. He advocated treatment by sealing up the surface and shutting off oxygen from the part.

Von Sehlen[3] recommends a zinc-ichthyol paste containing ichthyolammon, two parts; starch and zinc, a.a., twelve parts; and vaseline, twenty-five parts. Tomony[4] records a case which had existed three years in spite of all ordinary treatment, but which was cured within six weeks after the application of ichthyol.

Perrin[5] shows that eczema pilare of the upper lip is due to the staphylococcus aureus, and so advises frequent shaving followed by washing with a supersaturated solution of boracic acid in alcohol. The patient should avoid catching cold.

Du Castel[6] relates a case of eczema which occurred in a healthy man after remaining for some time in a cesspool.

The hypodermic injection of the ammonio-citrate of iron is recommended in cases of eczema due to anæmia. The results of this treatment in the hands of Pellizzari[7] have been specially favourable in the case of female patients.

In eczema of infants Marfan[8] recommends lavage of the intestine and stomach with boiled water, and the internal administration of salol. Locally, he first applies a weak solution of corosive sublimate for four days, and then he orders an ointment containing oxide of zinc, sulphur, and lanoline.

**Savill's Disease.**—Savill[9] contributes a further article on his epidemic skin disease. He considers that the disease belongs to the eczematous group of skin diseases; but he gives a table of the means of making a diagnosis from ordinary eczema. Although undoubtedly parasitic in origin, he found that the application of perchloride of mercury always did harm. The *Lancet*[10] and *British Medical Journal*[11] both publish leading articles on this disease, and discuss the various epidemics which have occurred. The Local Government Board is now undertaking an inquiry into the causes of the outbreaks of this disease. Lees[12] gives a clinical lecture on this condition, and relates a case. He believes that it is due to the diplococcus discovered by Savill.

**Dermatitis Herpetiformis.**—Nammack[13] describes a case of the disease which occurred in a girl aged 19, suffering from consumption. She presented typical grape-like clusters of vesicles, which itched intensely. Under treatment for her lung trouble the eruption disappeared, but a relapse was expected. The patient herself noticed exacerbation of the eruption corresponding to emotional excitement or anger. Hallapeau[14] and Tete examined the urine of a patient during an attack of this disease, and found it loaded with urates; they isolated a product which has all the characteristics of a toxin. Experimental inoculation with the alkaloid produced a specific ulceration at the point of puncture.

**Lupus.**—A discussion on lupus at the British Medical Association was opened by Leloir,[15] who considered lupus to be the most frequent form of cutaneous tuberculosis. He states that the entrance of the bacillus occurs either by (1) by direct inoculation, (2) indirect inoculation by continuity from deeper tuberculous foci, (3) inoculation by way of the lymphatics, (4) infection of hæmetic origin, and (5) infection by inheritance. The two first methods are much the most frequent. Walker[16] considers that the ulceration of lupus is distinctly produced by coccal contamination, its character, too, is more catarrhal than ulcerative. Gottheill[17], in considering the diagnosis of lupus, states that the disease always begins in youth before the age of puberty; he further regards lupus erythematosus, as non-tubercular, and therefore in no way connected with lupus. He recommends the use of the currette combined with a 10 per cent. solution of corrosive sublimate in alcohol. Taylor[18] reviews the various methods of treating lupus employed during the last twenty years, and gives a brief account of his personal experience founded on about 250 cases of the disease. He arrives at the following conclusions:—

1. That excision should no longer be practised except when lupus attacks a finger or a toe, and has proved rebellious to other treatment.

2. That scraping followed by the actual cautery is the most satisfactory.

3. That linear scarification is the remedy, *par excellence*, for extensive lupus of the face, where scarring has to be avoided.

Hutchinson[19] relates a case of lupus occurring in a scrofulous patient, and acknowledges the tubercular nature of the disease. Walsh[20] records a case of lupus occurring in the scars produced by a seton. Elsenberg[21] found that applications of parachlorophenol have an effect similar to that of tuberculin, while being free from the dangers of the latter drug. He uses an ointment containing equal parts of parachlorophenol, lanoline, vaseline, and starch. The ointment is applied to the patch, and allowed to remain on for ten or twelve hours. It is then removed and salicylic or iodoform ointment applied. After two days the parachlorophenol is again used. It is rather painful, and a few seconds after the application the patch of lupus becomes pale, and the surrounding area gets red and infiltrated. The patch quickly heals under a scab, but the cure does not appear to be permanent. Brocq[22] found that the use of iodoform to a patch of lupus exedens on the face caused the disappearance of some patches of lupus erythematosus on the hands. This latter eruption he considered was due to the absorption of toxins from the original patch.

**Erysipelas.**—Cavafy,[23] in a clinical lecture on erysipelas, states that the facial form is only contagious when a strong predisposition to the disease is present. He does not consider it necessary to isolate such cases, but treats them in the general medical wards. Anders[24] gives the following directions for treatment. Proper attention must be paid to the diet, but stimulants are rarely necessary. Iron is the best drug, but quinine and pilocarpine are also useful in some cases. Locally, the application of corrosive sublimate is the most efficacious after scarification, but in cases of erysipelas migrans, the germicide should be injected hypodermically just beyond the edge of the inflamed area. Gage[25] recommends as a new treatment the application of carbolic gauze soaked in a (one in twenty) carbolic acid solution, and covered with a sheet of mackintosh tissue. It is changed every four hours. Eleven cases so treated quickly recovered. Gaston[26] strongly recommends the hypodermic injection of carbolic acid, and uses the following formula—℞, acid carbolic, ʒ 1; glycerine, ʒ 3; aqua, ʒ 4, ♏. One syringeful to be injected daily. It is necessary to repeat the injection daily for three days, but not after this. In one case only was there no improvement. A case of periodically recurring erysipelas is recorded, which remained free from any attack for five years after this injection treatment. Whalon[27] recommends guiacol, but finds that in some cases the application of the pure drug causes pain; he, therefore, dilutes it with alcohol or olive oil, and applies from twenty to thirty minims at a time. The *New York Medical Journal*[28] reviews the local antiseptic treatment of this disease, and specially recommends the use of a 1 per cent. etherial solution of mercuric chloride in the form of a small spray. This, however, often produces a painful smarting sensation, but cuts short the disease within twenty-four hours. The combination of tartaric acid with the mercurial spray was found to act as a sedative. Felsenthal[29] recommends superficial and deep scarification, followed by the rubbing in of a (60 per cent.) ichthyol ointment or solution; antiseptic dressings are then applied. The results, however, are no better than those following less severe methods of treatment. Roger[30] insists that the pneumonia, which occurs during an attack of erysipelas, is due to pneumonococci, and that the development of the pneumonococci, in the course of erysipelas, is explained by the fact that the resistance of the patient has been lowered by absorption of the toxic poisons of the streptococcus. Netter[31] found pneumonococci in the saliva of six out of twelve cases recovering from erysipelas, and states that they were found more frequently even than streptococci.

**Urticaria.**—Abrahams[32] recommends the treatment of acute urticaria by hydrochlorate of pilocarpine. In adults he gives a hypodermic injection of from one-sixth to one-half of a grain, and in children he gives, by the mouth, one-twentieth to one-sixth of a grain in distilled water. The dose is repeated every day, or every other day. It is necessary to watch the patient for half an hour after the injection. His results are excellent. McBrayer[33] relates the case of a child aged two and a-half years, who experienced immediate relief after this method of treatment. Lanz[34] advocates the internal administration of ichthyol; within twenty minutes of taking a 20-drop dose of this drug all symptoms disappeared, and there was no recurrence of the trouble. The *Therapeutic Gazette*[35] has a leading article on the treatment of this disease, in which the various methods of treatment are referred to. Afzelius[36] records a case of urticaria pigmentosa in a child aged six months; the spots were most numerous on the upper part of the back and on the back of the right leg. It is the first recorded case in Sweden. Garrod[37] showed a child aged three years with a typical eruption of urticaria pigmentosa. Some of the spots had existed two and a half years.

**Parasitic Diseases.**—Leslie Roberts,[38] in a paper before the British Medical Association, states that the small amount of progress in the knowledge of the vegetable hair parasites is due to the want of appreciation of the relative importance of the different methods of research. He thinks that the parasite should be studied from the physiological point of view as well as from the microscopical and bacteriological. He then criticises the work of Sabouraud, and points out that it is absurd to endeavour to distinguish species by differences in the mycelia alone, since the appearance of cultivations can be altered by changes of temperature. He thinks that the soil, and the mode in which the fungus attacks its host, have a considerable share in producing microscopic varieties. The ectothritic formation of spores suggests a direct animal origin of the disease. Sabouraud[39] again reviews his work on trichophytosis, and urges the existence of several varieties of the trichophyton tonsurans. Wickham[40] considers that all work on this subject must include observations on these three facts—clinical characters, histology, and bacteriology; any work, without these, is valueless; pure lesions (those which have not been treated) should alone be investigated. He divides the parasites of tinea tonsurans into (1) microsporon Ardouini, in which the spores are small, and there is an absence of mycelium, and in which the development of the parasite in the hairs takes place from above downwards; (2) the trichophyton megalosporon which he subdivides into (*a*) trich. meg. endothrix, in which the spores are large and situated inside the hair, and there is a development of strong mycelial threads; and (*b*) the trich. meg. ectothrix, in which the mycelial threads are found in fragile chaplets, and the parasites are outside the hair. In proof of the distinctiveness of these different forms, he states that, in the same epidemic, and on one head, one always finds the same parasite. Raymond[41] remarks on the increase in the number of cases of tinea in Paris, and urges the necessity for the indoor treatment of children suffering from this disease; he considered the proposed establishment of a school for 310 children, so affected, as quite inadequate, and urges the foundation of dispensaries, containing 100 beds each, in different parts of Paris. Hutchinson[42] recommends the application of tincture of iodine for cases of tinea of the non-hairy parts, and the alternate application of the two following ointments when the scalp is affected: (1) Acid salicylic, 10 grs.; acid chrysophanic, 25 grs.; ichthyol (30 per cent.); andvaseline, 1℥. (2) A 20 per cent. ichthyol ointment. As a rule, his cases are cured within three weeks. McElligott[43] treats sycosis menti in the following way: A strip of lint soaked in vinegar is applied and covered with oil silk; this is removed in 12 hours, when the part is dressed twice daily with lint soaked in a 1 in 1,200 solution of mercuric chloride. After four days the dressing is only applied twice a week, and no further treatment is required after three weeks. Behrend[44] considers that the cure of pityriasis versicolor cannot be obtained by using simply parasitic agents, since the spores are situated in the interior of the follicle. He considers that chrysorobin gives the best results, as it determines desquamation not only of the skin, but also of the walls of the follicles. Hodara[45] describes the occurrence of trichorrhesis amongst the young women of Constantinople. It is manifested by the development of greyish points at the ends of the hairs, and by the division of the hair into several filaments. The swellings are smaller than those found in trichorrhesis nodosa, and are formed at the angle of the bent hair. A small rod-shaped bacillus was found, so the disease must be considered a contagious one. Feulard[46] recommends the following ointment for the treatment of acarus in children with delicate skins: Lard, 750 grains; balsam of Peru, 75 grains; naphthol, 15 grains. The ointment is applied at night, and the next morning the skin is well lathered with soap and is dusted with powdered starch.

[1] Brit. Med. Jour., Sep. 22nd, 1894, p. 645; Brit. Jour. of Derm., Sep., 1894, p. 275; Med. Record, Nov. 24th, 1894, p. 671. [2] Brit. Jour. of Derm., Sep., 1894, p. 282. [3] Monastshefte fur Derm., 19th Band, 1894. [4] Lancet, Dec. 22nd, 1894, p. 1,479. [5] Med. Week, Dec. 7th, 1894, p. 599. [6] Ditto, July 20th, 1894, p. 346. [7] Lancet, Dec. 22nd, 1894, p. 1,503. [8] Glas. Med. Jour., Nov., 1894. [9] Lancet, Sep. 29th, 1894, p. 733. [10] Ditto, ditto, p. 745, and ditto, Sep. 22nd, 1894, p. 703. [11] Brit. Med. Jour., Sep. 22nd, 1894, p. 652, and ditto, Sep. 29th, 1894, p. 713. [12] Clin. Journ., Oct. 31st, 1894, p. 14. [13] New York Med. Journ., Sep. 1st, 1894. [14] Med. Week, Aug. 17th, 1894. [15] Brit. Med. Journ., Sep. 8th, 1894, p. 517, and Brit. Journ. of Derm., Oct., 1894, p. 298. [16] Ditto, ditto. [17] Int. Med. Mag., Oct., 1894, p. 661. [18] Brit. Journ. of Derm., Nov., 1894, p. 345. [19] Clin. Journ., Dec., 1894, p. 97. [20] Brit. Journ. of Derm., Dec., 1894, p. 365. [21] Journ. Bi-Mens, July, 1894, p. 325; Central Fur Chir., Sep, 8th, 1894, p. 854; Med. Week, July 20th, 1894, p. 348. [22] New York Med. Journ., Sep. 16th, 1894, p. 347. [23] Clin. Journ., Oct. 31st, 1894, p. 18. [24] Ther. Gaz., July 16th, 1894, p. 447; Quarterly Med. Journ., Oct., 1894. [25] New York Med. Rec., Nov. 3rd, 1894. [26] Ther. Gaz., July 16th, 1894. [27] Ditto, ditto. [28] New York Med. Journ., Nov. 3rd, 1894, p. 574. [29] Ther. Gaz, July 19th, 1894, p. 490. [30] Med. Week, July 20th, 1894, p. 345. [31] Ditto, July 27th, 1894, p. 355. [32] Med. Rec., Sep. 15th, 1894 p. 342. [33] Ditto, Nov. 2nd, 1894, p. 564. [34] Med. Week, Sep. 7th, 1894, p. 440. [35] Ther. Gaz., Aug. 15th, 1894, p. 536. [36] Brit. Med. Journ., ep., Oct. 20th, 1894, p. 61. [37] Brit. Journ. of Derm., Dec., 1894, p. 369. [38] Ditto, Nov., 1894, p. 355; Brit. Med. Journ., Sep. 29th, 1894, p. 685. [39] Med. Week, Aug. 10th, 1894, p. 374. [40] Brit. Journ. of Derm., Oct., 1894, p. 308. [41] New York Med. Journ., Sep. 1st, 1894, p. 289. [42] Clin. Journ., Nov. 21st, 1894, p. 66. [43] Lancet, Oct. 27th, 1894. [44] Med. Week, Nov. 2nd, 1894. [45] Ditto, Oct. 26th, 1894. [46] New York Med. Journ., Oct. 20th, 1894, p. 512.

